# 

**Published:** 1895-05

**Authors:** 


### CONTENTS—MAY, 1895.—VOL. X., NO. 11.

ORIGINAL COMMUNICATIONS—

Reflections upon the Increase of Mental Unsoundness, D. R. Wallace, M. D., LL. D., Waco . . . . . . . . . . . . . . . . . . . . 569
Discussion on Dr. Wallace's paper . . . . . . . . . . . . . . . . 582
Morphine Narcosis. J. F. Eaves, M. D., Millican . . . . . . . . . . 585
Relationship of Gonorrhœa to Diseases of the Female Pelvic Organs. Russell Caffery, M. D., San Antonio . . . . . . . . . . . 587
Rhinological Don'ts. Edward J. Bermingham, A. M., M. D., New York . . . . . . . . . . . . . . . . . . . . . . . . . . . . . . . 589
The Dangers of Potent Drugs in the Hands of Careless Invalids. E. B. Jackson, M. D., Houston . . . . . . . . . . . . . . . . . . 593

SOCIETY NOTES—

The Late Meeting of the Texas State Medical Association . . . . . 595

EDITORIAL—

Death of Dr. Cupples . . . . . . . . . . . . . . . . . . . . . . . 605
Biographical—Dr. P. C. Coleman . . . . . . . . . . . . . . . . . . 608
Dr. Wallace's paper . . . . . . . . . . . . . . . . . . . . . . . . 609

MEDICAL NEWS AND MISCELLANY—

Numerous Interesting Paragraphs . . . . . . . . . . . . . . . . . 611
Death of Dr. Cupples . . . . . . . . . . . . . . . . . . . . . . . 615
Medical Advertising and the Mails . . . . . . . . . . . . . . . . 620
A Texas Doctor in Luck . . . . . . . . . . . . . . . . . . . . . . 622

BOOK NOTICES— . . . . . . . . . . . . . . . . . . . . . . . . . . 623

PUBLISHERS' NOTES . . . . . . . . . . . . . . . . . . . . . . . . . 626

